# The impact of supplementary narrative-based information on colorectal cancer screening beliefs and intention

**DOI:** 10.1186/s12885-015-1167-3

**Published:** 2015-03-21

**Authors:** Lesley M McGregor, Christian von Wagner, Gemma Vart, Wing Chee Yuen, Rosalind Raine, Jane Wardle, Kathryn A Robb

**Affiliations:** 1 Cancer Research UK Health Behaviour Research Centre, Department of Epidemiology and Public Health, University College London, London, UK; 2 Department of Applied Health Research, University College London, London, UK; 3 Medical Research Council/Chief Scientist’s Office, Social and Public Health Sciences Unit, Institute of Health and Wellbeing, University of Glasgow, Glasgow, UK

**Keywords:** Colorectal, Cancer screening, Faecal Occult Blood test, Beliefs, Narratives, UK

## Abstract

**Background:**

The potential benefits of colorectal cancer screening are limited by low uptake. This study tested whether providing narrative accounts of the colorectal cancer (CRC) screening experience positively affected beliefs about CRC screening and intention to be screened.

**Methods:**

4125 adults aged 45-59.5 years, from three general practices in England, were randomised to be sent the standard information on CRC screening or the standard information plus a narrative-based leaflet describing CRC screening experiences. Both groups were asked to complete and return a questionnaire on beliefs about CRC screening after reading the study materials. Between-group differences on responses were assessed with t-tests. A mediation analysis then addressed the mediating role of CRC screening beliefs on the group and intention relationship.

**Results:**

Relative to the standard information group (n = 590), the standard information plus narrative leaflet group (n = 631) showed higher perceived vulnerability to CRC, higher perceived test response efficacy, a stronger belief that the screening test would provide peace of mind and less disgust with the test procedure. There were no between group differences on perceived self-efficacy or the understanding that the screening test should be done in the absence of symptoms. Respondents who received the additional narrative leaflet reported significantly higher CRC screening intentions than respondents who received the standard information only. Controlling for the CRC screening beliefs reduced the effect of group on intention to non-significance.

**Conclusions:**

An additional narrative leaflet had a positive impact on beliefs about CRC screening which led to stronger screening intentions.

**Electronic supplementary material:**

The online version of this article (doi:10.1186/s12885-015-1167-3) contains supplementary material, which is available to authorized users.

## Background

Colorectal cancer (CRC) is the second most common cause of cancer death in the US and Europe [1,2]. Early detection through population-based screening is considered key to reducing CRC mortality rates [3,4].

In England, a national CRC screening programme was introduced in 2006 and is offered biennially to all men and women between 60 and 74. It is based on the home-based guaiac faecal occult blood test (FOB test) with follow-up colonoscopy investigation for abnormal results. Trials of this screening approach in the UK have shown a 10-13% reduction in CRC mortality in intention-to-treat analyses, rising to 27% among those who returned the FOB test kits [5,6].

Delivery of the CRC screening programme uses strategies known to maximise screening participation such as automated mailing of the FOB test to homes [7], pre-notification [8], and reminders (e.g. [9,10]). However, CRC screening uptake remains around 54%. There is also a strong socioeconomic gradient, with uptake ranging from 35% in the most deprived areas to 61% in the least deprived areas in England [11]. Therefore, further strategies that can increase uptake of CRC screening across all socioeconomic deprivation groups are important.

Several beliefs about CRC screening have been identified within the literature as barriers to uptake [12]. According to the Extended Parallel Process Model (EPPM) [13], increasing perceived personal risk of CRC should lead to positive behavioural change (i.e. screening), if accompanied by belief in one’s own ability to complete the test correctly (self-efficacy), and belief that doing the test can reduce the risk of CRC (response efficacy). The empirical literature also suggests more specific barriers to using the FOB test. Collecting stool samples can be perceived as unpleasant or disgusting and may put people off completing the test [14,15], while a lack of bowel symptoms can also cause people to believe that screening is not necessary for them [16,17]. In contrast, believing that screening can provide peace of mind can increase screening motivation [16,18]. Strategies to address these barriers and motivators have the potential to positively influence intentions to be screened.

Health information material is typically presented in a didactic format but the inclusion of narrative based presentations has recently been suggested to enhance engagement with the topic, which in turn can help promote adherence to recommended health behaviours (e.g. [19]). Narratives convey information through characters telling stories of relevant events, and are considered a natural and easily processed form of communication [20,21]. They are thought to aid positive behaviour change by reducing counter-arguing, facilitating mental imagery, and providing role models of behaviour [20-22]. Use of narrative based information as a strategy to reduce barriers to cancer prevention and early detection behaviours has shown some positive results. For example, a video showing breast cancer survivor stories resulted in a reduction in the number of perceived barriers to mammography and stronger intention to attend breast cancer screening [23]. Similarly, the inclusion of a tailored narrative to online information about CRC screening was associated with a reduction in the perceived impact of barriers, increased personal risk of getting CRC and increased intention to be screened [24]. The inclusion of narrative-based information was associated with an increase in attendance at colonoscopy [25] and flexible sigmoidoscopy [26].

Using narrative-based information to reduce barriers to FOB screening specifically has not yet been investigated. Improving uptake within the constraints of the NHS CRC screening programme requires a strategy that is easy to implement and has minimal cost implications. This study therefore assessed the impact of a narrative-based information leaflet that could be mailed out with the current pre-notification invitation letter and information booklet. The leaflet was not designed to be an alternative to current information materials but rather a supplementary resource to address key beliefs about CRC screening that can act as barriers or motivators to uptake with the purpose of enhancing intention to be screened.

Including selected narratives about FOB testing and various outcomes was hypothesised to increase feelings of CRC risk and self-efficacy for completing the test correctly, to increase the perceived benefits of screening (reduce the chances of dying from CRC, provide peace of mind), and reduce specific barriers (disgust with the test procedure and the belief that screening is only for those with symptoms). Together, the expected positive impact of the narrative leaflet on the above beliefs was hypothesised to translate into an increase in intention to be screened. Specifically, it was hypothesised that participants receiving the supplementary narrative information would report higher scores on feelings of risk, self-efficacy, perceived benefits and intentions, and lower scores on barriers than participants receiving the standard information.

## Methods

### Design and study population

Men and women aged between 45 and 59.5 years, and registered at one of three general practices in England (two in London and one in rural North West England, serving areas of mixed deprivation levels), were mailed information on CRC screening and invited to complete a postal questionnaire. The age range ensured that while people were approaching the screening age, they had not yet participated in the screening programme and therefore had no direct experience of the screening process which could influence their beliefs and responses to the information material. Potential invitees with known colorectal health problems or who were considered too unwell or unsuitable for participation were identified by the GP practice and excluded. Eligible adults (n = 4125) were randomly assigned to one of two groups (‘standard information’ (SI); n = 2067, and ‘standard information + narrative leaflet’ (SI + N); n = 2058) using Random Allocation Software [27]. Those invited were clustered by household before randomisation to ensure co-habiting individuals were assigned to the same group to minimise cross-over effects.

### Required sample size

The sample size calculation was based on the results of a previous study using narrative information on breast cancer screening attitudes [23]. Assuming α = 0.05 and power (1-β) = 0.90 the number needed was 684 (342 per group). A conservative response of around 20% was anticipated due to experience with recent surveys of similar design and so approximately 4000 people were invited to take part.

### Procedure and materials

Participants were sent a covering letter with the screening information, the questionnaire, and a Freepost return envelope. The covering letter was signed by the individual’s general practice and explained the purpose, process and voluntary nature of the study. The information resembled the official invitation sent out as part of the CRC screening programme in England, i.e. an NHS branded envelope containing a sample screening invitation letter and the standard 16 page information booklet (A5 size), entitled ‘Bowel Cancer Screening: The Facts’, published by the NHS CRC Screening Programme [28]. The standard information booklet includes text on what CRC is, what the screening process involves, and the aim, risks and benefits of CRC screening, as well as signposting for further information and support. Those randomised to the SI + N group received an additional narrative leaflet developed for this study entitled ‘Bowel Cancer Screening: People’s Stories’ (see Additional file 1). Participants were informed that return of a completed questionnaire was an indication of their consent to take part in the study.

A reminder letter, signed by the general practice, was sent to all non-responders approximately 4 weeks after the initial study invitation with another copy of the questionnaire, information material and return envelope. Data collection began in June 2012 and was completed by January 2013. Ethical approval was granted by the National Research Ethics Service (NRES) Committee, North East - Northern and Yorkshire.

### Development of a narrative-based barrier reducing leaflet

The narrative information material was designed to be a practicable addition to a large national screening programme and was therefore presented in a tri-fold, A4 leaflet (see Additional file 1). For the content, 20 volunteers (12 females; 8 males) were interviewed about their CRC screening experience: 8 had CRC diagnosed, 9 had benign polyps removed, and 3 had a negative FOB test result. The majority of volunteers were ‘white British’ or ‘white other’ (n = 16). An expert panel read through the interview transcripts and selected the quotes and stories for inclusion in the narrative leaflet, ensuring a mix of gender and ethnicity.

The selected quotes and stories predominantly focused on the psychological and physical outcomes of the decision to take part in screening (e.g. feeling ‘lucky’ to have had cancer picked up early) [29], recognition of vulnerability to CRC, the considered ability of the test to reduce the chance of death from CRC, self-efficacy in relation to test completion, and importance of getting ‘peace of mind’. Quotes also noted overcoming the feeling that the test is disgusting or that it is only for people with symptoms of CRC. The narrative information was mainly presented as first-person quotes with two additional summarised stories describing the full CRC screening experience. The overall tone of the leaflet was positive as a consequence of the overwhelmingly positive narratives provided. Although doubts about doing the test were often described, they were overcome and all participants were extremely supportive of CRC screening.

A photograph of each volunteer who provided their CRC screening narrative was added to the leaflet, with their consent. This was intended to enhance the reader’s identification with others who have successfully completed the screening test, and produce a more vivid message, factors linked to behavioural intention [30]. ‘Real’ people were used to legitimise the quotes and stories used.

An early version of the leaflet was discussed with experts in social marketing and their advice was integrated into a further iteration of the leaflet e.g. to use more natural photographs and only one quote to illustrate each point. The amended version was then the subject of a telephone interview with 3 lay people (2 male) with an interest in health research, who commented on content, design and layout. This was then followed by focus groups (n = 6; 3 male and n = 4; 1 male) with individuals in the targeted participant age range (45-59 years) for this study, recruited from a local community group. The feedback was positive but suggestions were also made to improve the leaflet e.g. to add ‘Bowel Cancer Screening’ to the title of the leaflet and to simplify the design of the front page. A subsequent version was then sent to those who had been interviewed for the narrative content and telephone interviews (n = 14) confirmed the final leaflet design. The final version was also reviewed by a health promotion officer and 4 academics using the Suitability and Comprehensibility Assessment of Materials (SAM + CAM) questionnaire and was deemed to be of a ‘superior standard’ [31].

### Measures

The questionnaire contained questions adapted from previous studies and assessed beliefs about CRC that may act as barriers or motivators to screening (i.e. perceived vulnerability, self-efficacy, test response efficacy, peace of mind, disgust with the procedure, and symptoms as a pre-requisite to screening), as well as intention.

An introductory question was asked to encourage or remind the participant to read the information material sent to them prior to beginning the questionnaire, ‘Have you read the orange booklet, ‘Bowel Cancer Screening: The Facts’ found inside the NHS envelope?’ The intervention group were additionally asked the same question in relation to the narrative leaflet. For both questions responses were on a 4 point scale ranging from “No” to “I have read it all more than once”.

Future intention to participate in bowel cancer screening was measured by a single item, ‘Imagine you have just turned 60 and have received the bowel screening test kit (FOB test kit) in the post. Doing the test involves taking small amounts of your stool (poo) on three different days and putting them on the FOB test kit. Realistically speaking, how likely are you to do this’. Responses were on a 4-point scale ranging from “Definitely not” to “Yes, definitely”.

Single items were included to assess the following beliefs in relation to screening: Perceived vulnerability: ‘If I never do the FOB screening test, I would feel very vulnerable to bowel cancer’ [32]; Self-efficacy: ‘I would be confident that I could do the FOB test correctly’ [15]; Response efficacy: ‘Doing the FOB test would reduce my chances of dying from bowel cancer’ [33]; Peace of mind: ‘Doing the FOB test would give me peace of mind’ [18]; Disgust with the test procedure: ‘Doing the FOB test would be disgusting’ [15]; Symptom absence: ‘I would only do the FOB test if I had symptoms of bowel cancer’ [15]. All responses were on a scale from “Strongly disagree” to “Strongly agree”: a 4 point scale for all items except perceived vulnerability (5-point scale including a midpoint of ‘Not sure’).

Respondents were asked to give their age, gender and ethnicity. Socioeconomic deprivation was assessed with three questions referring to current living arrangements (1 point for not owning own home), education (1 point for having no formal qualifications), and car ownership (1 point for not owning a car). Deprivation scores ranged from 0 (least deprived) to 3 (most deprived) [34].

### Data analysis

SPSS Statistics for Windows, Version 20.0 was used to analyze the data. Independent samples t-tests were used to assess between group differences in each of the key CRC beliefs and intention to be screened. Correlations were used to assess associations between beliefs and intention. The potential mediating effects of CRC screening beliefs on intention were analysed using the INDIRECT macro for SPSS. Output allowed review of the direct effect of group on each belief, of each belief on intention, and the effect of group on intention when controlling for beliefs. Effects reported are unstandardized. INDIRECT also allowed a comparison of the indirect effect of each belief to be reviewed and used the bootstrapping method with bias-corrected confidence estimates [35]. The 95% confidence interval of the indirect effects was obtained with 5000 bootstrap examples [36].

## Results

A total of 4124 people were invited to take part in this study (SI: 2067; SI + N: 2057) between July and September 2012. Completed questionnaires were returned by 1256 people (SI: 606, 29.3%; SI + N: 650, 31.6%) giving an overall response rate of 30.5%. The between group difference in response rate was not significant (p > 0.05). Participants whose self-reported age was different from the data obtained from their general practice (n = 35) were removed from analysis. The final sample was therefore n = 1221 with n = 590 in the SI group and n = 631 in the SI + N group. The two groups were similar in terms of socio-demographic characteristics (see Table 1). Of those who answered the introductory question(s), the majority self-reported that they had read at least some of the information materials provided (SI: 96%; SI + N: 94% and 90% for ‘The Facts’ booklet and narrative leaflet respectively).

**Table 1:** **Participant characteristics**

Variable	SI	SI + N
	*n = 590*	*n = 631*
Age		
Mean (SD)	51.94 (4.31)	51.80 (4.16)
*Missing*	-	-
Gender (n (%))		
Female	334 (56.6%)	353 (55.9%)
Male	256 (43.4%)	278 (44.1%)
*Missing*	-	-
Ethnicity (n (*%*))		
White	513 (86.9%)	547 (86.7%)
Non-white	75 (12.8%)	82 (13.0%)
*Missing*	2 (0.3%)	2 (0.3%)
Socioeconomic deprivation score (n (*%*))		
0 (least deprived)	326 (55.3%)	362 (57.4%)
1	145 (24.6%)	147 (23.3%)
2	71 (12.0%)	77 (12.2%)
3 (most deprived)	19 (3.2%)	16 (2.5%)
*Missing*	29 (4.9%)	29 (4.6%)

Note: *SI* = Standard information only group, *SI* + *N* = Standard information and narrative leaflet group.

### Beliefs about screening

Mean scores for each of the questions on beliefs about CRC screening, and the results of the between group comparisons, are presented in Table 2. As hypothesised, participants in the SI + N group reported higher scores on perceived vulnerability (p = .045) and response efficacy (p = .008) than the SI group. They also reported stronger beliefs that doing the FOB test would provide peace of mind (p = .002) and less disgust about doing the test (p = .007). Differences between groups in terms of their self-efficacy to complete the FOB test correctly (p = .055) or in their understanding that symptoms were not required for screening participation (p = .165) were not significant.

**Table 2:** **Post information beliefs by group**

Construct	Question	Scale*	SI (n = 590)*M (SD)*	SI + N (n = 631)*M (SD)*	Result
Perceived vulnerability	If I never do the FOB screening test, I would feel very vulnerable to bowel cancer	1-5	3.13 (.99)	3.25 (1.06)	t(1208) = -2.00, p = .045
Self-efficacy	I would be confident that I could do the FOB test correctly	1-4	3.29 (.55)	3.36 (.57)	t(1208) = -1.92, p = .055
Response efficacy	Doing the FOB test would reduce my chances of dying from bowel cancer	1-4	3.17 (.66)	3.27 (.66)	t(1201) = -2.64, p = .008
Peace of mind	Doing the FOB test would give me peace of mind	1-4	3.22 (.61)	3.33 (.61)	t(1199) = -3.15, p = .002
Disgust	Doing the FOB test would be disgusting	1-4	1.92 (.75)	1.81 (.71)	t(1204) = 2.69, p = .007
Symptom absence	I would only do the FOB test if I had symptoms of bowel cancer	1-4	1.67 (.70)	1.60 (.71)	t(1196) = 1.39, p = .165

*Higher scores = more agreement.

### Intention

Participants in the SI + N group had stronger intentions to complete the test kit (M = 3.71, SD = 0.53) than the SI group (M = 3.64, SD = 0.57), t(1208) = -1.98, p = .048. Table 3 shows the shift from uncertainty towards a positive response in those who received the additional narrative leaflet.

**Table 3:** **Responses to Intention question by group**

Group	1. Definitely not %	2. Probably not %	3. Yes probably %	4. Yes definitely %
SI (n = 582)	0.3	3.6	27.5	68.6
SI + N (n = 628)	0.6	1.8	24.0	73.6

Note: *SI* = Standard information group, *SI* + *N* = Standard information + narrative leaflet group.

Intention correlated significantly with each of the beliefs and in the anticipated direction. Table 4 shows the correlation matrix with beliefs in order of highest association with intention. All beliefs were also significantly inter-correlated.

**Table 4:** **Correlation matrix of the study variables**

		1	2	3	4	5	6	7
1	Intention	1						
2	Self-efficacy	.395*	1					
3	Peace of mind	.379*	.421*	1				
4	Symptom absence	-.339*	-.358*	-.261*	1			
5	Response efficacy	289*	.338*	.457*	-.222*	1		
6	Disgust	-.251*	-.299*	-.193*	.276*	-.133*	1	
7	Perceived vulnerability	.233*	.115*	.331*	-.106*	.231*	-.089*	1

*correlation is significant at the p < 0.01 level (2-tailed); Note: n = 1157 (listwise).

### The mediating role of key beliefs

**Figure 1:**
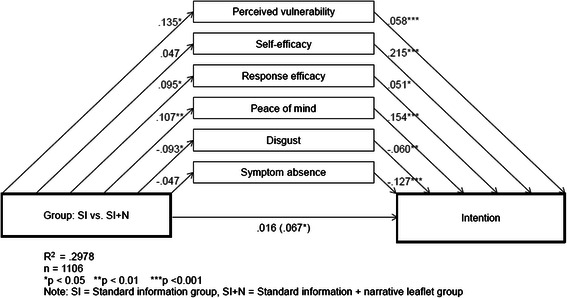
**Parallel multiple mediator model**. Parallel multiple mediator model showing relationships between group, intention and beliefs. Unstandardised beta coefficients show direct effects.

To help understand the process through which the narrative leaflet influenced beliefs and intention, a mediation analysis was conducted. Multiple regression analyses were carried out to confirm the direct effect of group on each belief, each belief on intention and group on intention (controlling for beliefs). The parallel multiple mediator model is presented in Figure 1. The model was based on a sample of 1106 people (SI: 529; SI + N: 577) and accounted for 29.8% of the variance in intention.

Age, gender, ethnicity and deprivation were included as control variables in the model. Older age was associated with stronger intention (B = .008, t(1095) = 2.55, p = .014); women were more likely to intend to do the CRC screening test than men (B = .066, t(1095) = 2.34, p = .019), and a higher socioeconomic deprivation level was associated with lower intention (B = -.061, t(1095) = -3.43, p < .001). There was no association between ethnicity and intention (p = .135). The relationship between group and beliefs, beliefs and intention, and group and intention were retained when controlling for gender, age, deprivation and ethnicity. The direct effects are presented in Table 5 (and in Figure 1).

**Table 5:** **Regression coefficients, standard errors and model summary information for the parallel multiple mediator model depicted in Figure** **1**

	B^a^	SE	t^b^	p-value
Direct effect of Group on each belief				
Perceived vulnerability	.135	.062	2.19	.029
Self- efficacy	.047	.033	1.44	.150
Response efficacy	.095	.040	2.38	.017
Peace of mind	.107	.036	2.97	.003
Disgust	-.093	.044	-2.13	.034
Symptom absence	-.047	.041	-1.14	.254
Direct effect of each belief on Intention^c^				
Perceived vulnerability	.058	.014	4.04	.000
Self- efficacy	.215	.030	7.24	.000
Response efficacy	.051	.024	2.13	.033
Peace of mind	.154	.028	5.44	.000
Disgust	-.060	.021	-2.93	.004
Symptom absence	-.127	.022	-5.67	.000
Total effect of Group on Intention				
Group	.067	.032	2.08	.038
Direct effect of Group on Intention^d^				
Group	.016	.028	.578	.564

^a^Unstandardised coefficient, ^b^df = 1095; ^c^Holding all other beliefs and Group constant; ^d^Holding beliefs about screening constant.

Importantly, the direct effect of group on intention reduced and became non-significant when the belief variables were controlled for, indicating that the leaflet affected intention through beliefs (see Table 5 and Figure 1).

Indirect effects were assessed using bias corrected bootstrap confidence intervals. Perceived peace of mind, vulnerability to CRC, anticipated disgust, and perceived test efficacy all mediated the effect of group on intention. The SI + N group had stronger intentions to be screened as a result of their tendency to, in order, i) more strongly believe that screening will provide peace of mind (B = .0167, CI = .0061, .0319), ii) perceive themselves as more vulnerable to CRC if they do not have screening (B = .008, CI = .0014, .0181), iii) consider the screening test as less disgusting (B = .006, CI = .0007, .0143), and iv) more strongly believe that screening would reduce their chances of dying from CRC (B = .005, CI = .0004, .0136) compared to the SI group. Examining the confidence intervals for each mediator contrast showed that the indirect effect of peace of mind was significantly stronger than the indirect effect of response efficacy (B = -.012, CI = -.0275, -.0009). No other significant differences were found suggesting that each of the other mediator indirect effects were of comparable strength.

## Discussion

The addition of a narrative leaflet to standard information material resulted in positive changes to beliefs and increased intention to be screened. A mediation analysis indicated that the effect of the leaflet on intention was mediated largely through its effects on anticipated peace of mind, perceived vulnerability to CRC, perceived disgust, and perceived efficacy of the test. Previous research successfully demonstrating the use of narrative information have typically presented stories via an online source, allowing for audio/video formats to be easily employed and the content to be tailored to the needs of the individual reader (e.g. [23,24]). However, these results show that even a generic, narrative-based paper leaflet mailed to participants along with standard material has the potential to influence beliefs about CRC screening that can act as barriers or motivators to uptake, and in so doing increase intention to be screened.

Health behaviour change models, such as the Extended Parallel Process Model (EPPM) [13] and the Health Belief Model (HBM) [37], highlight the importance of key beliefs in the decision to perform a particular behaviour. Such beliefs tend to centre round the extent of the threat to health, the benefits in performing the proposed behaviour, one’s own confidence to perform the behaviour, as well as additional barriers to completion. This study suggests that narrative-based information focusing on these constructs can change beliefs and enhance behavioural intentions, a pre-requisite to behavioural action.

According to the EPPM, awareness of the seriousness of a health threat (severity and susceptibility) is essential and must be accompanied by high self-efficacy and high perceived efficacy of the behaviour to motivate behaviour change. In the absence of high efficacy, the reaction is likely to be defensive [13]. In the present study, the threat was a future diagnosis of CRC and the behaviour to be elicited was screening, indexed in this study with intention to be screened. As cancer is perceived to be a serious disease, severity was not explicitly addressed. However, we did see an increase in perceived vulnerability to CRC in the group who received the additional narrative which contributed to increased intention to be screened. However, it is important to note that the mean score for perceived vulnerability remained close to an ‘unsure’ response. This result suggests room for further investigation of this component within the narrative leaflet, potentially generating a more pronounced impact on intention.

It was anticipated that reading about other people who have successfully completed the FOB test would enhance self-confidence in the ability to complete the test [38]. However, self-efficacy for test completion was relatively high in this sample overall and so our manipulation may have been too subtle to elicit the desired impact. Rather than an implied demonstration of test completion, a more explicit description may have had a stronger effect. The leaflet mainly included experience and outcome narratives in relation to screening, but process narratives may have been more effective at dispelling uncertainty as to how the test is completed [29]. The benefit of providing detailed instructions with the FOB test kit has previously been demonstrated in a study of CRC screening uptake rates [39]. Future research should address more precisely which components of narrative content and, additionally, which role models are most influential on intentions and subsequent behaviour.

This study had limitations. The percentage of people who responded was only 30.5% introducing potential selection bias. Closer inspection of the intention data shows that even within the SI group the majority of participants said they would probably (28%) or definitely (69%) be screened in the future, and belief scores were generally positive. Additionally, variation in socioeconomic deprivation was minimal in both groups. Therefore, we may not have reached people who had decided to not take part and may have benefited most from the supplementary leaflet.

Differences on the belief scores and intention were observed between the two groups but it remains to be seen if the positive, yet small, results from the inclusion of the narrative leaflet would have an impact on actual screening behaviour. Further research would be needed to clarify this.

Participants were aged 45-59.5 years old and it is not known whether the pattern of results would be the same among people currently eligible for CRC screening in England (i.e. aged 60-74 years). We chose to include this slightly younger sample of people approaching the eligible screening age rather than include people of screening age whose beliefs and responses to the information may have been influenced by their own CRC screening experience.

The narrative leaflet was designed to address selected barriers to screening, but the research topic itself is often considered socially unacceptable (e.g. [40]). This may have undermined participation and the importance of disgust may have been underestimated. The multiple task requirements may also have played a part in the low response rate obtained. Participation involved at least two tasks: reading a 16 page document (the standard information booklet) as well as the narrative-based leaflet for the SI + N group, and then completing a questionnaire.

This study tested intentions to be screened and not actual screening behaviour. However, the strength of intention to take part in CRC screening has been shown to be an important predictor of screening behaviour, [12] and so the increase in intention resulting from the inclusion of an additional narrative leaflet could have positive public health implications, and should be investigated in future research.

## Conclusions

This study describes the initial assessment of newly developed narrative information material for possible use in the NHS CRC screening programme. The study demonstrated broadly positive results suggesting that a supplementary narrative leaflet is likely to positively affect beliefs associated with CRC screening intentions, which may in turn lead to increased CRC screening uptake. With no noted adverse effects from the narrative leaflet, its impact on behaviour could be investigated next.

## Additional file

Additional file 1: **Bowel Cancer Screening: People’s Stories.** A bmp file of the narrative leaflet designed for use in this study. This leaflet was sent with the information materials to those assigned to the Standard information + narrative leaflet (SI + N) group.

## Abbreviations

CRC: Colorectal cancer
EPPM: Extended Parallel Process Model
FOB test: Faecal Occult Blood test
GP: General Practitioner
HBM: Health Belief Model
NHS: National Health Service
NIHR: National Institute for Health Research
SPSS: Statistical package for the social sciences
SI: Standard information
SI + N: Standard information with additional narrative leaflet

## References

[1] American Cancer Society. Cancer facts and figures 2015. [http://www.cancer.org/research/cancerfactsstatistics/cancerfactsfigures2015/index]

[2] Ferlay J, Steliarova-Foucher E, Lortet-Tieulent J, Rosso S, Coebergh JWW, Comber H (2013). Cancer incidence and mortality patterns in Europe: Estimates for 40 countries in 2012. Eur J Cancer.

[3] Diaz JA, Slomka T (2012). State of the art review: colorectal cancer screening. Am J Lifestyle Med.

[4] World Health Organization (Europe). Action plan for implementation of the European strategy for the prevention and control of noncommunicable diseases 2012-2016 [http://www.euro.who.int/en/health-topics/noncommunicable-diseases/cancer/publications/2012/action-plan-for-implementation-of-the-european-strategy-for-the-prevention-and-control-of-noncommunicable-diseases-20122016]

[5] Libby G, Brewster DH, McClements PL, Carey FA, Black RJ, Birrell J (2012). The impact of population-based faecal occult blood test screening on colorectal cancer mortality: a matched cohort study. Br J Cancer.

[6] Scholefield JH, Moss SM, Mangham CM, Whynes DK, Hardcastle JD (2012). Nottingham trial of faecal occult blood testing for colorectal cancer: a 20-year follow-up. Gut.

[7] Church TR, Yeazel MW, Jones RM, Kochevar LK, Watt GD, Mongin SJ (2004). A randomized trial of direct mailing of fecal occult blood tests to increase colorectal cancer screening. J Natl Cancer Inst.

[8] Libby G, Bray J, Champion J, Brownlee LA, Birrell J, Gorman DR (2011). Pre-notification increases uptake of colorectal cancer screening in all demographic groups: a randomised controlled trial. J Med Screen.

[9] Tseng DS, Cox E, Plane MB, Hla KM (2001). Efficacy of patient letter reminders on cervical cancer screening: a meta analysis. J Gen Intern Med.

[10] Wagner TH (1998). The effectiveness of mailed patient reminders on mammography screening: A meta-analysis. Am J Prev Med.

[11] von Wagner C, Baio G, Raine R, Snowball J, Morris S, Atkin W (2011). Inequalities in participation in an organized national colorectal cancer screening programme: results from the first 2.6 million invitations in England. Int J Epidemiol.

[12] Power E, Van Jaarsveld CH, McCaffery K, Miles A, Atkin W, Wardle J (2008). Understanding intentions and action in colorectal cancer screening. Ann Behav Med.

[13] Witte K (1992). Putting the fear back into fear appeals: The extended parallel process model. Commun Monogr.

[14] Chapple A, Ziebland S, Hewitson P, McPherson A (2008). What affects the uptake of screening for bowel cacner using faecal occult blood test (FOBt): a qualitative study. Soc Sci Med.

[15] Jones RM, Devers KJ, Kuzel AJ, Woolf SH (2010). Patient-reported barriers to colorectal cancer screening: a mixed-methods analysis. Am J Prev Med.

[16] van Dam L, Korfage IJ, Kuipers EJ, Hol L, van Roon AHC, Reijerink JCIY (2013). What influences the decision to participate in colorectal cancer screening with faecal occult blood testing and sigmoidoscopy?. Eur J Cancer.

[17] Palmer CK, Thomas MC, von Wagner C, Raine R (2014). Reasons for non-uptake and subsequent participation in the NHS Bowel Cancer Screening Programme: a qualitative study. Br J Cancer.

[18] Robb KA, Solarin I, Power E, Atkin W, Wardle J (2008). Attitudes to colorectal cancer screening among ethnic minority groups in the UK. BMC Public Health.

[19] Kim HS, Bigman CA, Leader AE, Lerman C, Cappella JN (2012). Narrative health communication and behavior change: the influence of exemplars in the news on intention to quit smoking. J Commun.

[20] Green MC (2006). Narratives and cancer communication. J Commun.

[21] Kreuter MW, Green M, Cappella J, Slater M, Wise M, Storey D (2007). Narrative communication in cancer prevention and control: A framework to guide research and application. Ann Behav Med.

[22] Miller-Day M, Hecht ML (2013). Narrative means to preventative ends: a narrative engagement framework for designing prevention interventions. Health Commun.

[23] Kreuter MW, Holmes K, Alcaraz K, Kalesan B, Rath S, Richert M (2010). Comparing narrative and informational videos to increase mammography in low-income African American women. Patient Educ Couns.

[24] Dillard AJ, Fagerlin A, Cin SD, Zikmund-Fisher BJ, Ubel PA (2010). Narratives that address affective forecasting errors reduce perceived barriers to colorectal cancer screening. Soc Sci Med.

[25] Jensen JD, King AJ, Carcioppolo N, Krakow M, Samadder NJ, Morgan S (2014). Comparing tailored and narrative worksite interventions at increasing colonoscopy adherence in adults 50-75: a randomized controlled trial. Soc Sci Med.

[26] Wardle J, Williamson S, McCaffery K, Sutton S, Taylor T, Edwards R (2003). Increasing attendance at colorectal cancer screening: testing the efficacy of a mailed, psychoeducational intervention in a community sample of older adults. Health Psychol.

[27] Saghaei M (2004). Random allocation software for parallel group randomized trials. BMC Med Res Methodol.

[28] Bowel Cancer Screening. The Facts [http://www.cancerscreening.nhs.uk/bowel/publications/the-facts.html]

[29] Shaffer VA, Zikmund-Fisher BJ (2012). All stories are not alike: a purpose-, content-, and valence-based taxonomy of patient narratives in decision aids. Med Decis Making.

[30] Dillard AJ, Main JL (2013). Using a health message with a testimonial to motivate colon cancer screening: associations with perceived identification and vividness. Health Educ Behav.

[31] Helitzer D, Hollis C, Cotner J, Oestreicher N (2009). Health literacy demands of written health information material: an assessment of cervical cancer prevention materials. Cancer Control.

[32] Dillard AJ, Ferrer RA, Ubel PA, Fagerlin A (2012). Risk perception measures’ association with behavior intentions, affect, and cognition following colon cancer screening messages. Health Psych.

[33] Rawl S, Champion V, Menon U, Loehrer PJ, Vance GH, Skinner CS (2001). Validation of scales to measure benefits of and barriers to colorectal cancer screening. J Psychosoc Oncol.

[34] Wardle J, McCaffery K, Nadel M, Atkin W (2004). Socioeconomic differences in cancer screening participation: comparing cognitive and psychosocial explanations. Soc Sci Med.

[35] Preacher KJ, Hayes AF (2004). SPSS and SAS procedures for estimating indirect effects in simple mediation models. Behav Res Meth Ins C.

[36] Preacher KJ, Hayes AF (2008). Asymptotic and resampling strategies for assessing and comparing indirect effects in multiple mediator models. Behav Res Methods.

[37] Rosenstock IM (1966). Why people use health services. Milbank Meml Fund Q.

[38] Bandura A (1977). Self-efficacy: toward a unifying theory of behavioral change. Psychol Rev.

[39] Hewitson P, Ward AM, Heneghan C, Halloran SP, Mant D (2011). Primary care endorsement letter and a patient leaflet to improve participation in colorectal cancer screening: results of a factorial randomised trial. Brit J Cancer.

[40] Reeder AI (2011). “It’s a small price to pay for life”: faecal occult blood test (FOBT) screening for colorectal cancer, perceived barriers and facilitators. The New Zeal Med J.

