# Supplementary figures and images for: The impact of supplementary narrative-based information on colorectal cancer screening beliefs and intention
Source: BMC Cancer. 2015 Mar 21;15:162. doi: 10.1186/s12885-015-1167-3 (PMC4397889; doi:10.1186/s12885-015-1167-3)

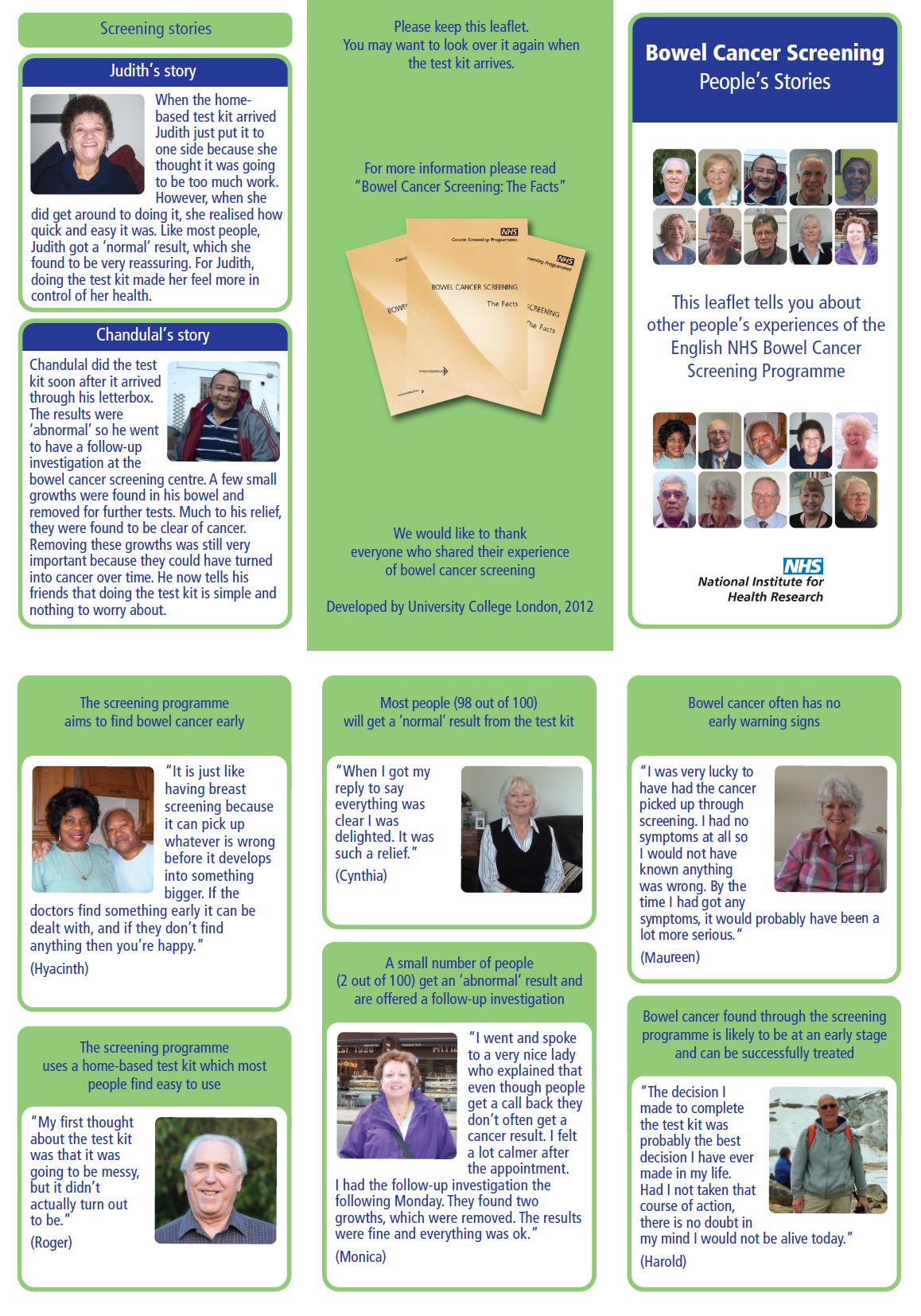

Supplement: Additional file 1: — Bowel Cancer Screening: People’s Stories. A bmp file of the narrative leaflet designed for use in this study. This leaflet was sent with the information materials to those assigned to the Standard information + narrative leaflet (SI + N) group. [file 12885_2015_1167_MOESM1_ESM.tiff]
